# A co‐designed framework to support and sustain patient and family engagement in health‐care decision making

**DOI:** 10.1111/hex.13054

**Published:** 2020-04-27

**Authors:** Tamara L. McCarron, Thomas Noseworthy, Karen Moffat, Gloria Wilkinson, Sandra Zelinsky, Deborah White, Derek Hassay, Diane L. Lorenzetti, Nancy J. Marlett

**Affiliations:** ^1^ The Department Community Health Sciences University of Calgary Calgary AB Canada; ^2^ O'Brien Institute for Public Health Calgary AB Canada; ^3^ Patient and Family Co‐Investigator; ^4^ Faculty of Nursing University of Calgary in Qatar Doha Qatar; ^5^ Haskayne School of Business Calgary AB Canada; ^6^ Health Sciences Library University of Calgary Calgary AB Canada

**Keywords:** capacity building, motivation, patient, patient participation, patient‐centred

## Abstract

**Background:**

Patient and family engagement in health care has emerged as a critical priority. Understanding engagement, from the perspective of the patient and family member, coupled with an awareness of how patient and family members are motivated to be involved, is an important component in increasing the effectiveness of patient engagement initiatives. The purpose of this research was to co‐design a patient and family engagement framework.

**Methods:**

Workshops were held to provide additional context to the findings from a survey. Participants were recruited using a convenience sampling strategy. Workshop data collected were analysed using a modified constant comparative technique. The core research team participated in a workshop to review the findings from multiple inputs to inform the final framework and participated in a face validity exercise to determine that the components of the framework measured what they were intended to measure.

**Results:**

The framework is organized into three phases of engagement: *why I got involved*; *why I continue to be involved*; and *what I need to strengthen my involvement.* The final framework describes seven motivations and 24 statements, arranged by the three phases of engagement.

**Conclusion:**

The results of this research describe the motivations of patient and family members who are involved with health systems in various roles including as patient advisors. A deeper knowledge of patient and family motivations will not only create meaningful engagement opportunities but will also enable health organizations to gain from the voice and experience of these individuals, thereby enhancing the quality and sustainability of patient and family involvement.

## 1 BACKGROUND

Without additional funding or the adoption of innovative approaches to service delivery, existing health‐care systems are unlikely to remain sustainable.^1^ Facing this challenge requires the meaningful involvement of multiple stakeholders across the entire health system, with particular emphasis on the recipients of care.^2^ Despite its importance, understanding ‘how’ best to involve patient and family members in decision making within all domains of health care remains unclear.^3^, ^4^ To date, research on effective and sustainable patient and family involvement is both scarce and has shown limited success in demonstrating or measuring impact.^5^, ^6^ Coupled with little guidance based on evaluative research on how to effectively involve patients, decision makers looking to draw transferable lessons to inform the design of meaningful patient engagement programmes and processes are largely absent.^7^, ^8^

Recognizing that individuals are motivated to satisfy needs and to maximize the value they receive becomes important to the effectiveness and sustainability of initiatives that involve patient and family members in health‐care decision making.^9^, ^10^, ^11^ Many health organizations in Alberta, Canada, such as Alberta Health Services, involve patient and family members in various roles, such as advisors or partners who participate in governance, in‐service health‐care delivery and other decision making activities. As described by Carman, higher levels of engagement are described by greater involvement, increased sharing of power and increased responsibility.^12^ Research on motivations is not a new area of discovery; however, understanding the motivations of stakeholders in health care, specifically why patient and family members are motivated to get involved and continue to stay involved, is largely unexplored.^13^, ^14^, ^15^, ^16^, ^17^, ^18^, ^19^, ^20^, ^21^, ^22^, ^23^ To inform our process, we used the market choice behaviour (MCB) theory, as a theoretical base, for its ease of use and as a recognized theory explaining how individuals are motivated to make choices.^10^, ^11^ The MCB theory draws from disciplines such as psychology and sociology and recognizes that individuals have limited time and resources, which compete with choices, such as getting involved or not.^10^, ^11^, ^14^, ^15^, ^24^, ^25^, ^26^, ^27^ The MCB theory identifies five values that independently influence an individual's choice behaviour: (a) functional; (b) conditional; (c) epistemic; (d) social; and (e) emotional.^11^ Since these values are independent of each other, the choice behaviour can be influenced by one or all five values.^10^, ^11^

In Canada, the term patient engagement is defined by the Canadian Institutes for Health Research, (CIHR) as the meaningful and active collaboration in activities such as governance and research (^28, p. 5^). ‘Patient’ is an overarching term, inclusive of individuals with personal experience of a health issue such as caregivers, family and friends.(^28, p. 5^). Acknowledging the concept of co‐design is still evolving, that co‐creation, co‐production and co‐design have evolved independently in different disciplines and are often confused and treated synonymously with one another, and we used key learnings, informed by the literature, to support how we worked together during this project.^29^, ^30^, ^31^, ^32^, ^33^, ^34^ For the purposes of this study, since we were exploring the motivations of patients at the higher levels of engagement, we felt co‐design was an appropriate way to work together. We define co‐design as the core research team, consisting of three patient co‐investigators and a researcher, working together, challenging themselves to work in partnership, to engage in shared leadership and shared decision making throughout the entire project.

The patient and family members, who acted as co‐investigators, were recruited using a convenience sampling strategy.^35^ An email poster was distributed to individuals within the authors’ personal network such as the Patient and Community Engagement Researcher (PaCER) Program, a programme introducing patients to qualitative research.^36^ In order to participate, individuals self‐identified as having experience and familiarity as a patient or family member with the health‐care system in Alberta were fluent in English, lived in Alberta and were over 18 years of age. Six individuals indicated interest, and three individuals were selected based on their area of interest and ability to commit to the entire project. All have considerable lived experience, either living with and/or supporting family members with serious chronic illness, were female and range in age from 45 to 78 years.

This manuscript reports on the findings from four regional workshops convened to provide additional context to a regional survey^37^ and interviews with patient and family members and describes the process used to develop a framework to explore and understand the motivations for patient and family engagement.

## 2 METHODOLOGY

**FIGURE 1:**
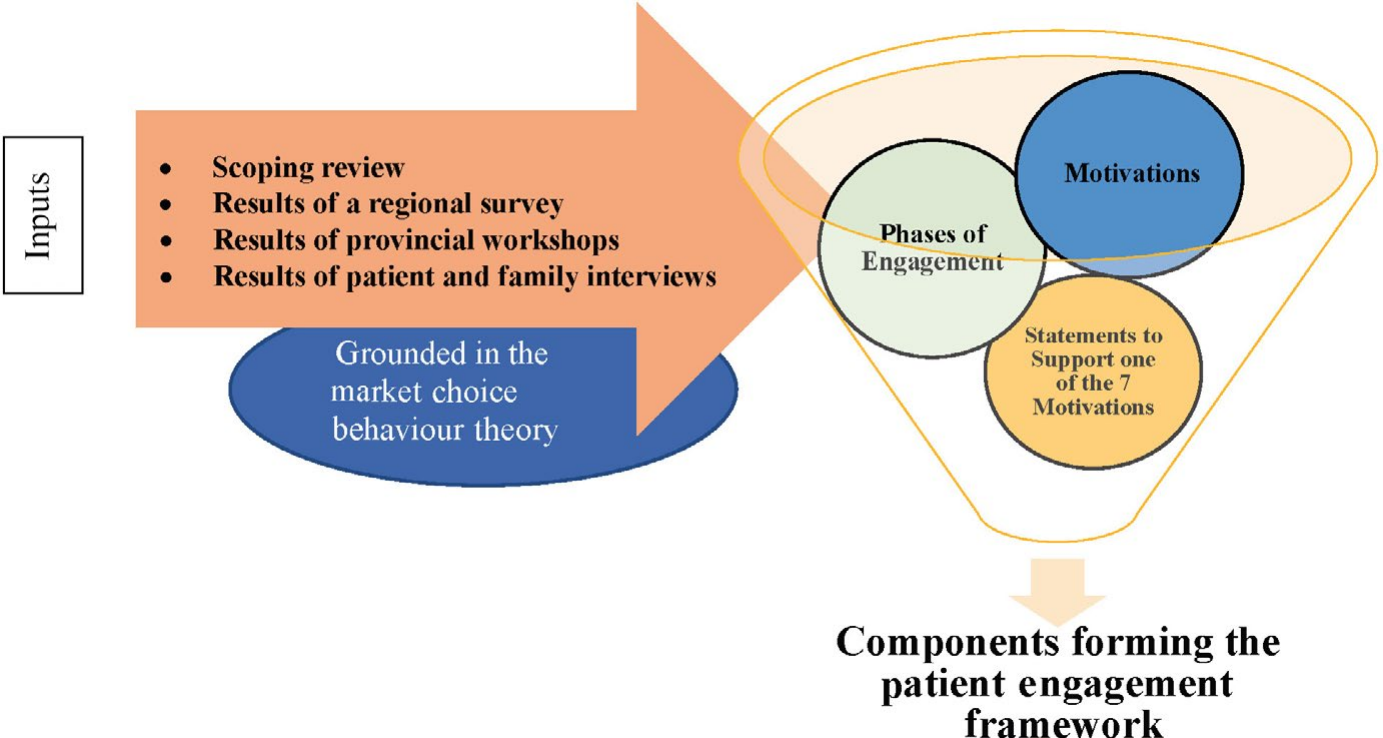
Inputs informing the patient and family engagement framework

A regional survey, informed by a scoping review^38^ and interviews with patient and family members, was analysed to understand the motivational factors of individuals who engage in health‐care decision making.^37^ Multiple inputs informed the patient and family engagement framework including the following: the results of a regional survey,^37^ interviews with patient and family members^37^ and four regional workshops (described below). See Figure 1. Workshop participants were recruited using a convenience sampling strategy,^35^ and data were analysed using a modified constant comparative technique.^39^ A final co‐design workshop was held with the core research team to review the data from the survey and workshops. Below, we describe the methods used to support the development of the final framework.

### 2.1 Recruitment for regional workshops

The purpose of the workshops was to inform the final framework by providing additional insight and understanding to the results of a regional survey.^37^ Two target populations of interest were identified: (a) patient and family members involved with health organizations, such as patient advisors; and (b) patient and family engagement professionals, such as those working in the health‐care system in Alberta. Patient and family engagement professionals were individuals who worked within health organizations and are responsible for designing and delivering engagement programmes in the region. Engagement professionals were included in the workshop to raise awareness of the findings from this research and assist with the contextualization and dissemination of the final framework.

A convenience sampling strategy was used to identify participants for the workshops.^40^ Patient and family members who had previously responded to a regional survey^37^ were contacted by email and asked whether they wished to participate in a future workshop. A comprehensive list of organizations with patient and family engagement programmes was identified during the survey phase,^37^ and individual organizations were contacted by phone to provide a patient engagement professional able to participate in one of the workshops.

### 2.2 Regional workshops

Four, 4‐hour workshops were held in June 2018 in Alberta, Canada. Locations were chosen to ensure a balanced participation from both urban and rural participants. The sessions were facilitated by the core research team. One month prior to the workshop, project consent forms were emailed to participants. An email was sent 2 weeks prior to the workshop with an agenda and overview of the research project. As participants arrived for the workshops, the core research team assigned individuals to each table. The composition of the groups was deliberately structured to limit the number of engagement professionals at each table, thus minimizing the power differentials between participants.^41^

### 2.3 Data collection and analysis

The workshops began with an overview of the research project, followed by highlights from the results of the scoping review^38^ and survey results.^37^ Participants then engaged in semi‐structured large‐group and within‐table discussions that were guided by a series of open‐ended questions, formulated to provide additional context to both the path of engagement, described during the patient and family interviews, (recruit, retain and sustain) and to the seven motivations identified as a result of the analysis of the regional survey.^37^ The sessions were audio‐recorded and analysed using a modified constant comparative method developed by Glaser.^39^ This required that the core research team complete a side‐by‐side comparison and analysis of the transcripts from each of the workshops to determine common themes.^39^, ^42^ Analysis continued until each theme was fully described and supported by data from the workshops.

### 2.4 Developing the patient and family engagement framework

On completion of the workshops, the core research team convened to synthesize the findings from the workshop and decide on the components and overall design of the framework. During this meeting, the draft of the framework was developed. Two weeks later, the patient and family co‐investigators participated in a face validity exercise where they attempted to subjectively determine the extent to which the components of the framework appeared to measure what they purported to measure.^43^ Any disagreements were resolved by achieving consensus through discussion. All meetings were facilitated by the primary author.

## 3 RESULTS

### 3.1 Characteristics of workshop participants

One hundred and sixty participants indicated an interest in participating. The research team grouped individuals based on their geographic proximity to one of four planned workshops and then randomly selected 36 participants to attend each event. Fifty‐three individuals participated in four workshops held in Calgary, Edmonton, Grande Prairie and Lethbridge between 4 June and 12 June 2018. Workshop participants consisted of patient or family members (68%, n = 36) and engagement professionals (32%, n = 17). Twenty‐five percent of participants were male (n = 13), and seventy‐five percent were female (n = 40). Sixty‐eight percent (n = 36) were from an urban centre (Calgary, Edmonton or Lethbridge), while thirty‐two percentage (n = 17) lived in a rural location. The majority of participants were aged 51‐65 (40%, n = 21) followed by 35‐50 (30%, n = 16), with the youngest aged 24 and the eldest being 88 years of age.

### 3.2 Part 1: Developing the components of the framework

#### 3.2.1 Understanding the phases of engagement

To support the development of the survey tool,^37^ patient and family members, actively involved in roles such as patient and family advisors, were previously interviewed to understand why these individuals were involved in these roles.^37^ Participants were asked to describe why they got involved, their perceived impact, contributions they had made and what they thought was needed to support their continued involvement. These interviews revealed a distinct path for engagement, defined by three phases: recruit, retain and sustain. To provide additional context and understanding of these three phases, we asked workshop participants to describe what each phase meant to them and provide an example. A summary of these findings is reported below.

#### 3.2.2 The ‘recruit’ phase

This phase defines the initial period when the individual makes the decision to get involved. Workshop participants felt that the initial recruitment phase was a complex process, unique to each individual and synonymous to establishing a new relationship (in this case a the relationship between patient and family member and the health system). The initial ‘courting’ stage occurred when an individual either personally experienced the health‐care system or experienced it on behalf of a loved one. This experience, positive or negative, in combination with the ability to find opportunities to become involved, such as the patient and family advisor role, is how participants described their initial engagement. ‘This process is a two‐way street. I have to have a desire to get involved and there has to be an opportunity to give input*’* (YEG‐04).

#### 3.2.3 The ‘retain’ phase

Participants describe this phase as why people stay involved. Participants shared the importance of being valued, or participating in something that was perceived as meaningful, as their reason for continuing in their role. Meaningful engagement was described by participants as the perception that the organization ‘valued their contributions’ (YYC‐03). Participants felt value was evident ‘when organizations report back’ (YYC‐03) and when they could see their contributions were ‘making an impact’ (YQU‐05). Others described meaningful as being ‘respected’ (YYC‐01) and having influence. ‘I wouldn't be involved if I didn't think I was making a difference’ (YEG‐03). Being ‘recognized’ helped to further solidify meaning by making individuals feel less like a ‘token’ and more ‘like a partner’ (YQL‐01). An opportunity to ‘be with others and to develop productive and mutually beneficial relationships’ helped people to feel welcome and to find meaning in their engagement (YQU‐03). Attending conferences and participating in other learning and development opportunities further enhanced the value for participants. ‘The learning is important. It allows us to start seeing the bigger picture and we start to feel like we are contributing to change’ (YQL‐03).

#### 3.2.4 The ‘sustain’ phase

Participants describe this phase as the support needed to continue their involvement. Many factors were described as key to the overall success of these patient and family engagement programmes. Participants describe this phase as including an adequate number ‘of people that could be called on’ (YEG‐02). When asked what participants felt was needed to support and strengthen patient and family engagement ‘buy‐in from decision makers’ (YQL‐05) was viewed as an important first step. Ultimately, participants felt their individual engagement would flourish when health ‘system[s] began valuing’ patient and family engagement (YYC‐06). Participants felt this would be evident when ‘resources to support these programs were adequate’ (YQL‐06); ‘ongoing strategies’ for patient and family engagement (YYC‐07) were developed; individuals were supported by ‘growing and developing their skills’ (YYC‐08); and regular ‘communication channels were established’ so individuals could better understand existing health‐care challenges and access information regarding engagement opportunities available to patients and families (YEG‐06).

#### 3.2.5 Understanding patient and family motivations

A provincial survey was previously administered to understand the motivations of patient and family members currently engaged with health systems.^37^ Seven motivations resulted from the analysis of the survey: *Self‐fulfilment*, *Improving Health care*, *Compensation*, *Influence*, *Learning New Things*, *Conditional* and *Perks*. To provide additional context and understanding to the seven motivations and corresponding statements (thirty‐nine in total), derived from the analysis of the survey,^37^ we asked workshop participants to describe what each motivation meant to them and provide an example. A summary of these findings is reported below.

#### 3.2.6 Self‐fulfilment

Participants described this motivation as the most complex and was primarily focused on an individual's desire to find purpose, to do something meaningful, to establish productive and rewarding connections; it was rooted in a sense of obligation and driven by the desire for the gratification provided by the opportunity itself such as participating in an activity to help others. Participants felt it was important that individuals were recognized for their contributions and that they felt there was a benefit from being involved. See Table 1.

**TABLE 1:** Understanding motivations by themes from the workshops from the perspective of the workshop participants

Motivation	Quote
*Self‐fulfilment*	
Purpose	[I am motivated to make] ‘the health care system better by making it easier for the next person’ (YYC‐10).
Altruistic	[This is an opportunity] ‘to do something good for others’ (YEG‐07).
Establishing relationships	[Being able to] ‘establish relationships with other people exposes you to new pieces of the puzzle’ (YQL‐12). [This experience] ‘provides an opportunity to meet people (provider and patient) with similar or different ideas and experiences’ (YYC‐11).
Obligation	[My life‐changing experience] motivated me to want ‘to pay it forward’ (YQL‐12).
Recognition	‘I feel like I have accomplished something and I am being recognized for these efforts’ (YQL‐10). [I am motivated] ‘by a feeling of gratification and having the ability to affect change and see the results of your efforts’ (YYC‐11).
*Improving Health care*	
Making a difference	‘To me this means it is making a difference at a system level, so the same thing won't happen again’ (YQL‐07). ‘My own personal experience was a huge motivator for me, my family member had issues and needed help in a negative medical situation’ (YQU‐04).
Speaking for others	‘This [opportunity] is like advocating for change and improvement while also advocating for patient and family centered care’ (YQL‐08).
*Compensation*	
Acknowledgement	[Compensation] ‘ensures people aren't being used for their professional background’ (YEG‐09). [Being compensated] ‘is a respectful acknowledgement that values my participation’ (YQU‐07).
Encouraging participation	[Even though I don't need to be compensated, it is important to include] ‘marginalized groups because it eliminates barriers’ (YQU‐08).
Choice	‘Paying for these services shouldn't be mandatory, I don't need it, but others might’ (YQL‐13).
*Influence*	
Access to senior health‐care administrators	[Influence is enhanced by] ‘being one of the movers or people with influence and when the people in power are asking you’ [to be involved] (YYC‐15). ‘Having access to influential folks not only give me more confidence it also reinforced that this was what was needed for change to occur’ (YQL‐19).
Partnership	‘Being treated like an equal and being part of this bigger group gives me the confidence to affect change’ (YQL‐16).
*Learning New Things*	
Being effective	‘Being able to do this work requires that you know about the system’ (YYC‐14).
Personal value	[Learning things] ‘allows me to think about things I may not have and learning increases my personal value” (YEG‐10).
Provides meaning	‘Learning keeps your mind stimulated, especially when you are retired and allows people to better understand issues’ (YQU‐10).
*Perks*	
Growing your network	[Travelling provides] ‘an opportunity to grow my network by interacting with like‐minded people across the province’ (YQU‐09).
Benefits	[Travelling] “is an exciting benefit that could be further leveraged by tying travel commitments with personal visits with family and friends’ (YEG‐12).
Rewards	[Travelling is a] ‘reward and an opportunity to learn new things’ (YQL‐18).
*Conditional*	
Flexibility	[I would appreciate more] ‘opportunities that are flexible’ (YEG‐16).
Support	‘Participating from home’ and ‘having IT support to get connected’ (YQU‐3).

Abbreviations: YEG, Edmonton; YGU, Grande Prairie; YQL, Lethbridge; YYC, Calgary.

#### 3.2.7 Improving Health care

Participants described this motivation as an individual's desire to fix the health‐care system by improving not only the quality and service delivery but also the internal culture such as the perceived attitudes of physicians towards patient and family members. Participants felt individuals were driven by a sense of advocacy and the ability to speak for others. See Table 1.

#### 3.2.8 Compensation

Compensation, in this context, was described by participants as beyond the payment of expenses and included monetary recognition that acknowledged an individual's time and talents. It was important to participants for comparable recognition to other individuals doing similar tasks. This motivation was embedded in how patient and family members measure the value of what they bring to the project and is seen as a mechanism to encourage participation. Participants overwhelmingly wanted to be given a choice as to whether they would receive compensation or not. See Table 1.

#### 3.2.9 Influence

Participants described this motivation as an individual's ability to impact decisions and to feel as though they were being heard and considered as a partner by other health‐care professionals. Participants felt this motivation was further enhanced when their perspective was acknowledged and valued by senior health professionals and others who could affect change. See Table 1.

#### 3.2.10 Learning new things

This motivation described an individual's desire to be exposed to new experiences and to have the opportunity to exercise knowledge, skills and abilities that might otherwise go unpractised. Learning enhanced participant's personal value and provided meaning to individuals outside of their formal role. Participants were passionate about this motivation and described it as key to their ability to be effective and an important enabler for change. See Table 1.

#### 3.2.11 Perks

Participants described this motivation similar to compensation, but rather than being financially rewarded, individuals were motivated by extra benefits such as expense reimbursements or opportunities to attend conferences. These experiences provided additional advantages to individuals such as growing and developing personal and professional relationships and connections. See Table 1.

#### 3.2.12 Conditional

The *conditional* motivation for participants was contingent on the specific situation faced by the individual. This motivation enhanced the choice, to participate or not, by increasing the perceived value to the individual. For example, this motivation was achieved when participants could participate in opportunities that were flexible and convenient to their schedules. Since these were unique to each situation, having multiple opportunities for people to get involved would satisfy individuals who are motivated by this value. See Table 1.

### 3.3 Part 2: Designing and validating the patient and family engagement framework

The core research team met to develop the framework components. The team reviewed the findings from the scoping review,^38^ the regional survey^37^ and the regional workshops to draw out any emerging themes to support the development of the framework. After examining the transcripts from the workshops, the three phases of engagement were renamed: *Why I got involved? (formerly recruit)*; *Why I continue to be involved? (formerly retain)*; and *What I need to strengthen my involvement? (formerly sustain)*.

Next, the team discussed the creation of visual framework to represent the stages of involvement. The team acknowledged the complexities in the decisions to not only get involved, but to continue being involved. They felt the final framework should present patient and family engagement as a journey. The team discussed how individuals could move through the framework, recognizing that the motivations at each phase would be unique to each individual, with one or all motivations potentially resonating with an individual at any given time.

Two weeks after this initial meeting, the patient and family co‐investigators participated in a face validation exercise where they were asked to take one of 39 statements, identified by the analysis of the survey, and assign them to one of seven motivations. The first round of face validation identified duplicates, which were later removed (n = 10) and ambiguous statements, which were either removed (n = 5) or renamed to provide further clarification (n = 2). Two days later, the face validation exercise was repeated with the remaining 24 statements. No additional duplicate or ambiguous statements were identified and there were no disagreements on the final results. See Table 2.

**TABLE 2:** Results of the co‐design process used to develop the patient and family engagement framework

Motivation	Statements identified from the survey	1st Round (Face validity)	2nd Round (Face validity)	Statements identified during co‐design workshop to support final framework	Phase of Engagement
Self‐fulfilment	I am making a difference	Duplicate	Removed	Removed	N/A
	A sense of purpose	Ambiguous	Renamed	This gives me a sense of purpose	Why I continue to be involved?
	I enjoy what I am doing		Kept	I enjoy what I am doing	Why I continue to be involved?
	Helping others	Ambiguous	Renamed	I get to help others	Why I continue to be involved?
	I feel I am making a difference	Duplicate	Removed	Removed	N/A
	I see the difference I am making		Kept	I see the difference I am making	Why I continue to be involved?
	I am supporting other patients		Kept	I am supporting other patients	What I need to strengthen my involvement?
	I have established important relationships		Kept	I have established important relationships	What I need to strengthen my involvement?
	I have improved patient experience		Kept	I have improved patient experience	What I need to strengthen my involvement?
Improving Health care	Improving the healthcare system	Duplicate	Removed	Removed	N/A
	To make healthcare better	Duplicate	Removed	Removed	N/A
	I want to improve the health care system		Kept	I want to improve the health care system	Why I got involved?
	To change the current culture of healthcare		Kept	To change the current culture of healthcare	Why I got involved?
	Helping to improve healthcare	Ambiguous	Renamed	I am helping to improve healthcare	Why I continue to be involved?
	I want to improve healthcare for myself and my family	Duplicate	Removed	Removed	N/A
	To speak for those who can't speak for themselves	Ambiguous	Removed	Removed	N/A
Compensation	I am getting paid	Duplicate	Removed	Removed	N/A
	I get paid		Kept	I get paid	Why I continue to be involved?
	Earning extra money	Duplicate	Removed	Removed	N/A
	It is an opportunity to make some extra money		Kept	It is an opportunity to make some extra money	Why I got involved?
	I receive payment		Kept	I receive payment	What I need to strengthen my involvement?
	I get to travel	Ambiguous	Kept	Removed	N/A
Influence	I am challenging the ‘norm’		Kept	I am challenging the ‘norm’	Why I continue to be involved?
	I am impacting decisions		Kept	I am impacting decisions	Why I continue to be involved?
	I am paving the way for others		Kept	I am paving the way for others	Why I continue to be involved?
	Others listen to me		Kept	Others listen to me	Why I continue to be involved?
	Communication between patients and health professionals have improved	Ambiguous	Removed	Removed	N/A
Learning new things	I get to learn new things		Kept	I get to learn new things	Why I got involved?
	Learning new things	Duplicate	Removed	Removed	N/A
	I learn new things		Kept	I learn new things	Why I continue to be involved?
	I continue to learn new things		Kept	I continue to learn new things	What I need to strengthen my involvement?
Perks	My expenses are paid		Kept	My expenses are paid	Why I continue to be involved?
	My expenses are paid	Duplicate	Removed	Removed	N/A
	I am able to travel		Kept	I am able to travel	What I need to strengthen my involvement?
Conditional	Your expenses are reimbursed	Ambiguous	Removed	Removed	N/A
	You can work from home		Renamed	I can work from home	What I need to strengthen my involvement?
	You attend an annual conference	Ambiguous	Removed	Removed	N/A
	The role could turn into a paid position	Ambiguous	Removed	Removed	N/A
	The commitment requires that you only attend four meetings per year		Renamed	My commitment is four meetings per year	What I need to strengthen my involvement?

**FIGURE 2:**
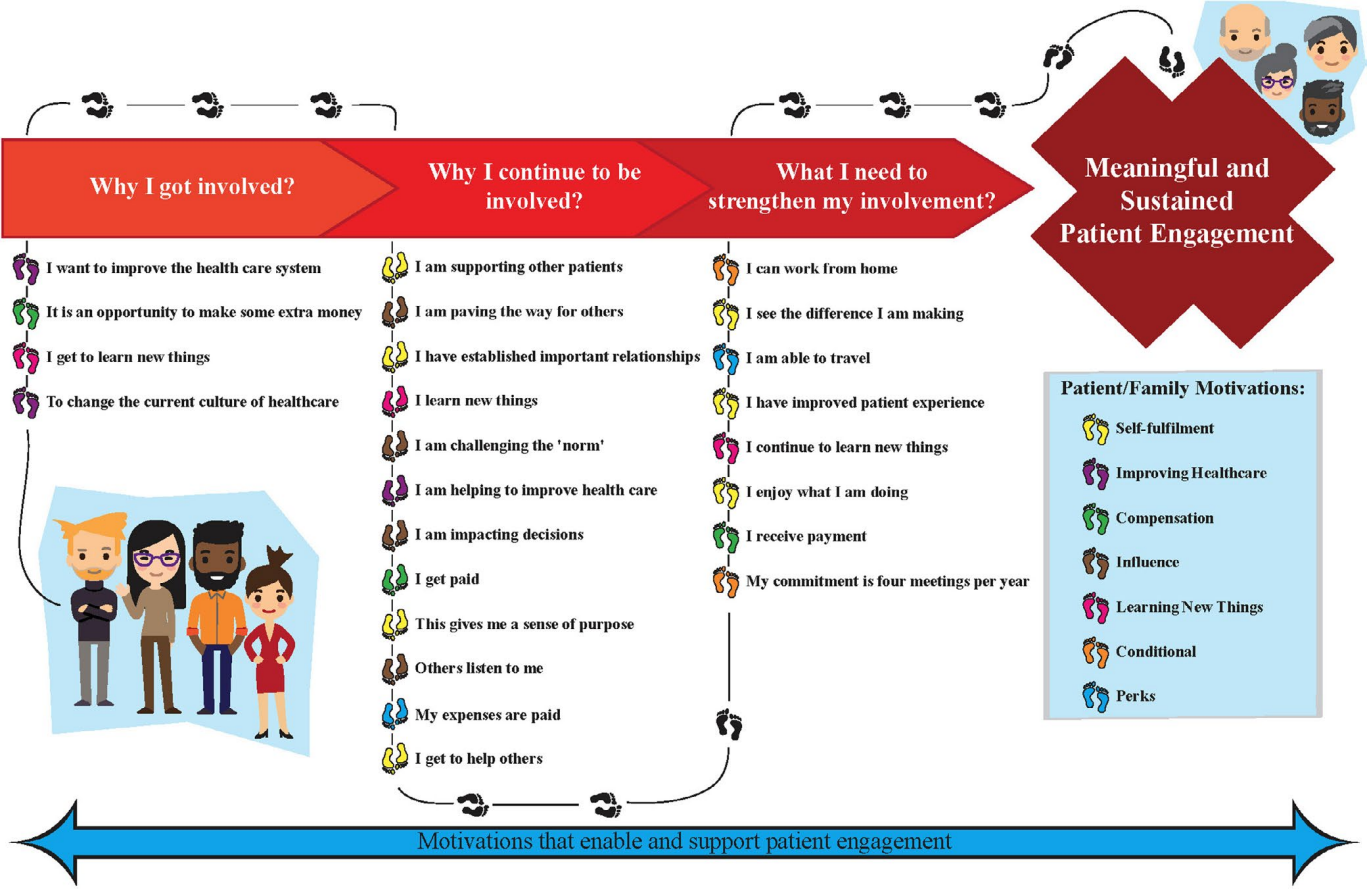
A Co‐designed framework to support and sustain patient engagement

The final framework describes seven motivations and 24 supporting statements, arranged by three phases of engagement. After organizing these statements by phase, *Why I got involved* includes 4 statements representing three of the seven motivations; *Why I continue to be involved* includes 12 statements which support five of the seven motivations; and *What I need to strengthen my involvement* includes 8 statements supporting five of the seven motivations. The final co‐designed patient and family framework can be found in Figure 2.

## 4 DISCUSSION

A framework for patient and family engagement, grounded in market choice behaviour theory and informed by the literature, a regional‐wide survey and four regional stakeholder workshops, was co‐designed from conception to completion with patient and family co‐investigators. This framework is based on the motivations of patient and family members and describes why these individuals make the choice to become and remain involved with health organizations. This framework defines 7 motivations: *Self‐Fulfilment*, *Improving Health care*, *Compensation*, *Influence*, *Learning New Things*, *Conditional and Perks* and describes how these motivations are important during three phases of patient and family engagement: why a person decides to get involved, why they continue to be involved and what is needed to strengthen their involvement. To the best of our knowledge, there is no known published research that explicitly presents a patient and family engagement framework that is based on patient and family motivations and incorporates a consumption value framework from marketing in a health‐care setting.

Practical implications of these findings are important for the future of patient and family engagement for three reasons. First, motivational research in health care focuses on understanding why individuals get involved but does not explore what motivates people to remain or sustain their involvement.^44^, ^45^, ^46^, ^47^ Designing to attract and retain these individuals is not only key to the success and sustainability of engagement initiatives, it is critical to fostering broader patient and family engagement. Second, this study is an example of how choice behaviour models such as the market choice behaviour theory can be used to better understand patient and family motivations. This opens an exciting area of research in understanding the choice decisions of patient and family members who are involved with health systems in various roles including patient advisors. Third, the workshops provide an enhanced understanding of the phases of engagement. Our findings suggest the importance of not only providing engagement opportunities but also promoting these activities so patient and family members can choose one that is right for them. Fourth, our findings suggest that individuals engage with health organizations to not only satisfy a specific need but to also maximize the value they receive. This reinforces our understanding of the role of choice and is an important component in the design and delivery of meaningful engagement programmes.

This research is unique in that we used a consumption value framework from marketing in a health‐care setting.^10^, ^11^ Our results confirm the findings from the motivational literature with some notable differences. Each of the 5 motivations, functional, conditional, social, emotional and epistemic, described in the MCB theory are represented in our results.^10^, ^11^ *Compensation* is an example of a functional value driven by the desire to satisfy a need, in this case being paid.^10^, ^15^, ^25^, ^27^ Individuals motivated by compensation are seeking to fulfil a financial need (removing barriers to participation) or to fulfil the need to be recognized by others (being paid is an acknowledgement of the patient/family member's contribution as a partner). *Learning New Things* is an example of an epistemic value. This motivation is driven by the need for an opportunity that provides novelty, arouses curiosity or satisfies knowledge.^10^, ^14^, ^15^, ^25^, ^27^ Individuals motivated by learning possess the desire for more knowledge and for self‐ improvement and are attracted by the novelty of this relatively ‘new role’ for patient and family members. *Conditional* is a unique motivation that has little to no value until an individual is placed in a situation that creates tension with the ability of the individual to maximize the value from their choice. In other words, an individual who is suddenly no longer able to drive will put higher value on opportunities that provide an option for being transported or being able to work from home. This means the value of this motivation is unique to the situation under consideration. *Improving Health care* is another example of a functional motivation. By wanting to make health care better, individuals are motivated to get the best performance and/or reliability from their health‐care system. *Influence* is an example of a social value. This motivation depends on the membership of the group, and individuals measure their status in relation to others within that group.^10^, ^48^ *Self‐fulfilment* is an example of an emotional motivation. This motivation recognizes that everything an individual does, no matter how noble and beneficial to others, has the most value, when the opportunity provides a perceived benefit to the individual themselves.^18^ *Perks* is an example of a social value that is associated with the symbolic meaning (and prestige) of being a patient or family advisor and member of the team. This value is realized when individuals attend conferences, requiring expense reimbursement for travel and registration fees.^49^ While these findings are encouraging, these results are preliminary, and more research is needed to further explore and unpack these findings.

This study has strengths and limitations. First, we must acknowledge that the presence of the primary researcher and the patient and family co‐investigators could have influenced the workshop results. All the individuals involved in this project are very passionate about patient and family engagement and could have inadvertently influenced the workshop dynamics or opinions of workshop participants. We attempted to minimize this with our facilitation techniques such as providing multiple opportunities for participants to provide input without the core research team's involvement. Second, when completing the face validity exercise, it is possible that the results could have been strengthened if individuals other than the core research team participated in the exercise. Third, the results were based on a sample of patient and family members volunteering in various roles within one health‐care system and therefore do not necessarily allow the findings to be generalized to the general population of volunteers or to other health‐care systems. Fourth, there was a lack of diversity among the patient and family co‐investigators. While recruiting the patient and family co‐investigators, we decided that the ability to commit to the entire length of the project (3 years) was important to provide consistency. We acknowledge that the patient and family co‐investigators were female, and a more diverse team may have resulted in additional insights that could have further enhanced the framework. Fifth, this work focuses on the motivations of patient and family members. We acknowledge the importance of involving diverse groups of stakeholders, including clinicians and other health‐care professionals, and encourage future studies to explore the motivations of these groups to support effective and meaningful involvement. Sixth, neither the primary researcher nor patient and family co‐investigators are qualitative researchers. A robust qualitative analysis using grounded theory or phenomenology may have provided additional context to our findings.

Given the current interest in patient and family engagement, coupled with the promising results of these findings, additional research in this area should be encouraged. The framework identifies the values of patient and family members that are significant to their decisions to get and stay involved. This information could prove to be exceptionally valuable to health systems wanting to involve patient and family members. Secondly, the framework lends itself to the development of strategies to support and sustain patient and family involvement. The MCB theory assumes individuals are making decisions for themselves about whether they will be involved or not, and on that understanding, motivations important to vulnerable and hard to reach populations are likely present within this framework, but more work should explore this hypothesis.^10^, ^11^, ^25^ Finally, the iterative process utilized in this study, with a team consisting of a researcher and three patient and family co‐investigators, and the involvement of key stakeholders at various stages of the project, could be a highly productive and meaningful model for those wishing to conduct health research and should be further explored.

## 5 CONCLUSION

While significant research exists that highlights the motivations of the public who choose to participate in decision making, a limited number of studies have explored these concepts within health care. This programme of work describes the process used to co‐design a patient and family engagement framework founded on a theoretical base, informed by the available evidence base, a regional survey, and deepened and clarified by a series of stakeholder workshops. The framework is comprised of seven motivations and three phases of patient and family engagement. As the roles of patient and family members in the context of health‐care decision making continue to evolve, the importance of effective and sustainable engagement programmes will become increasingly important. A deeper knowledge of patient and family motivations not only will help to create meaningful engagement opportunities for patient and family members but also will enable health organizations to gain from the experience of these individuals. While further research is needed to support diverse groups of stakeholders, the findings from this study have developed an understanding of how patient and family members are motivated to make decisions about their involvement.

## CONFLICT OF INTEREST

The authors declare they have no competing interests.

## ETHICAL APPROVAL

This study was approved by the Conjoint Health Research Ethics Board (CHREB) at the University of Calgary, Canada.

## Data Availability

The data sets used and/or analysed during the current study are available from the corresponding author on reasonable request.
